# Relationship Between Hypertension and Basilar Atherosclerosis in Chinese Han Population: A High-Resolution Magnetic Resonance Imaging Study

**DOI:** 10.3389/fcvm.2022.830664

**Published:** 2022-04-27

**Authors:** Feng Hu, Feng Lu, Huiling Xiao, Meixue Dong, Yan Xu

**Affiliations:** ^1^Department of Cardiovascular Medicine, The Second Affiliated Hospital of Nanchang University, Nanchang, China; ^2^Department of Cardiothoracic Surgery, The Second Affiliated Hospital of Nanchang University, Nanchang, China

**Keywords:** high-resolution magnetic resonance imaging, hypertension, basilar atherosclerosis, vulnerable plaque, Chinese

## Abstract

**Objective:**

To investigate the relationship between hypertension and basilar atherosclerosis evaluated by high-resolution magnetic resonance imaging (HR-MRI) in the Chinese Han population.

**Methods:**

High resolution-MRI vessel wall imaging was performed in selected 193 patients for various indications. Multivariable logistic regression models based on odds ratio (*OR*) with their associated 95% confidence interval (*CI*) were used to assess the relationship between hypertension and basilar artery (BA) plaque, moderate or severe stenosis of BA plaque, and vulnerable plaque. A linear regression model was used to assess the relationship between hypertension and BA plaque numbers.

**Results:**

Patients with hypertension had a higher proportion of BA plaque and vulnerable plaque as well as more number of enhancements of BA plaque and serious plaque compared with normotensive patients (all values of *p* < 0.05). Multivariable logistic regression analysis indicated that patients with hypertension had an increased risk for and more number of enhancements of BA plaque (adjusted-*OR*: 4.32, 95% *CI* 1.89–9.88, *p* < 0.001; adjusted-β: 0.55, 95% *CI* 0.14–0.96, *p* = 0.009, respectively) and had a higher proportion of moderate or severe stenosis of BA plaque and vulnerable plaque (adjusted-*OR*: 3.08, 95% *CI* 0.77–12.32, *p* = 0.111; adjusted-*OR*: 4.52, 95% *CI* 1.50–13.64, *p* = 0.007, respectively) compared with the normotensive group. Moreover, there was a saturation effect of age on the prevalence of BA plaque and vulnerable plaque.

**Conclusion:**

Hypertension was the independent risk factor of BA plaque and vulnerable plaque assessed by HR-MRI in the Chinese Han population.

## Introduction

Intracranial atherosclerosis is the main cause of stroke in the Chinese population, accounting for 46.6% of ischemic stroke cases, but the pathology is not well understood because it cannot be easily studied in living patients (1). When the basilar artery (BA), the most important vessel in the posterior circulation, has insufficient blood flow for the posterior circulation, patients would present with clinical signs and symptoms of brain ischemia (2).

Conventional techniques, such as computerized tomography angiography (CTA) and digital subtraction angiography (DSA), could reveal the abnormalities of the vascular lumen, but were unable to fully reveal the lesion of vascular wall and accurately distinguish the pathological characteristics of atherosclerotic plaque (3–8). In addition, the size, shape, signal intensity, and enhancement characteristics of atherosclerotic vulnerable plaque could not be recognized through luminal imaging (3–8). Interest in intracranial high-resolution magnetic resonance imaging (HR-MRI) is growing (4–8). Compared with conventional cavity imaging methods, HR-MRI could visualize the intracranial arterial disease and identify previously hidden mural lesions (7, 8).

The vulnerability of atherosclerotic plaque is closely related to the occurrence of stroke. There are five main criteria for vulnerable plaques: active inflammation, a thin cap with a macro-lipid core, endothelial denudation with platelet aggregation on the surface, rupture of the fibrous cap, and severe stenosis; The five secondary criteria for vulnerable plaques include superficial calcified nodules, yellow plaque under a vascular microscope, intraplaque hemorrhage (IPH), endothelial dysfunction, and positive remodeling (3–5).

Active inflammation, rupture of the fibrous cap, severe stenosis, intraplaque hemorrhage, and positive remodeling can be identified on HR-MRI (3–5). Inflammation plays a critical role in plaque initiation, progression, and disruption (9, 10). Histological analysis from atherosclerotic rabbits indicated that the degree of macrophage infiltration on the vascular wall was proportional to plaque enhancement on HR-MRI (11). Histological analysis indicated that gadolinium (Gd) enhancement of carotid plaque on HR-MRI was significantly associated with macrophages infiltration, neovascularization, and loose fibrosis in patients undergoing endarterectomy for carotid stenosis (12, 13). Therefore, inflammation could possibly be the underlying mechanism that causes plaque enhancement (3). Plaque contrast enhancement is associated with vascular active inflammation, and the high signal intensity on T1 weighted images (T1WI) strongly indicated the presence of intraplaque hemorrhage (14). When the plaque is large, a thin cap with a macro-lipid core and superficial calcified nodules could also be identified (3).

Based on the above background, this study aimed to assess the atherosclerotic plaque and vulnerable characteristics of BA based on HR-MRI and further analyze the risk factors of BA sclerosis in the Chinese Han population.

## Materials and Methods

### Participants

This was a retrospective study performed at the Second Affiliated Hospital of Nanchang University. Between January 2018 and January 2021, HR-MRI was performed on 231 individuals of consecutive Chinese Han population for various indications. Eligible participants were adults aged 18 years and older who voluntarily performed the HR-MRI. They had at least one of the following diseases: hypertension, diabetes, impaired cognitive function, ischemic stroke, or transient ischemic attacks. After excluding 38 subjects with confirmed or highly suspected non-atherosclerotic vasculopathy, such as arteritis, moyamoya disease, and dissection, 193 patients were included in this analysis. Written informed consent was obtained from all participants before each HR-MRI examination. The study was conducted in accordance with the Declaration of Helsinki and was approved by the Institutional Ethics Committees of the Second Affiliated Hospital of Nanchang University.

### Clinical Data Collection

Participants’ demographic characteristics [age, sex, and body mass index (BMI)], lifestyles (smoking and alcohol habits), medical history [hypertension, type II diabetes mellitus (T2DM), ischemic stroke, and transient ischemic attacks (TIA)], and medication usage were collected by trained research staff. Cognitive function was assessed using the Mini-Mental State Examination (MMSE) scale by a qualified medical professional (15).

### Laboratory Assay

Blood samples were collected using venipuncture after an overnight fast of at least 12 h. Blood biochemistries for fasting blood glucose (FBG), total cholesterol (TC), total triglyceride (TG), high-density lipoprotein cholesterol (HDL-C), low-density lipoprotein cholesterol (LDL-C), very low-density lipoprotein cholesterol (VLDL), plasma homocysteine (Hcy), serum uric acid and creatinine, blood urea nitrogen (BUN), and creatinine kinase-MB (CK-MB) were measured using automatic clinical analyzers at the clinical laboratory of the Second Affiliated Hospital of Nanchang University. The formula for estimated glomerular filtration rate (eGFR) used the Chronic Kidney Disease Epidemiology Collaboration (CKD-EPI) equation (16). Hypertension was defined as a seated systolic blood pressure (SBP) ≥ 140 mmHg and/or diastolic blood pressure (DBP) ≥ 90 mmHg on at least three separate clinic visits or previously diagnosed hypertension. The clinical classification in hypertension (Class I, II, and III) was defined as the 140/90, 160/100, and 180/110 mmHg threshold. The diagnosis of incident diabetes was defined as fasting glucose > 7.0 mmol/L and/or self-reported diabetes.

### HRMR Imaging Protocol

A 3.0 T MR scanner (GE Discovery 750W, America) with a 19-channel phased-array head-neck coil was used in this study. All patients underwent conventional T2 weighted imaging, diffusion-weighted imaging (DWI), three-dimensional (3D) time-of-flight (TOF) magnetic resonance angiography (MRA), and BA HR-MRI imaging, which included high resolution-T1 weighted imaging (HR-T1WI) plain and contrast material-enhanced black-blood magnetic resonance images, 3D-multiple overlapping thin slab angiography (MOTSA) sequences, and magnetization-prepared rapid gradient echo (MP-RAGE) sequences. Then, 3D-HRMRI images were acquired in a traversal plane to cover the major intracranial arteries identified on the TOF MRA. The HR-T1WI was obtained using a fast spin echo 3D technique (CUBE T1WI). The parameters were as follows: repetition time (TR) = 1,300 ms, echo time (TE) = 16 ms, field of view (FOV) = 18 cm, matrix = 320 × 320, zero-fill interpolation (ZIP) = 512, slice thickness = 0.6 mm, echo train length = 10, and number of excitation = 2. The display resolution was 0.35 mm × 0.35 mm × 0.6 mm. In total, 120 coronal slices covering the anterior and posterior circulation were acquired with a scan time of 15 min.

### Image Analysis of Basilar Artery Plaque

Images were independently analyzed by two experienced HR-MRI specialists blinded to the clinical data using visual inspection. The differences between the two observers were settled by consensus. Picture quality was assessed using a previously developed four-point scale (one = poor quality, two = sufficient quality, three = good quality, and four = excellent) (17). Images were assessed on all cross-sectional image slices with a score greater than or equal to two.

A plaque was defined as an eccentric wall thickening, and the thinnest part was estimated to be lesser than 50% of the thickest point by visual observation (18, 19). In our study, the number of plaques was only counted at the main trunk of BA. We defined the reference sites as the nearest plaque-free segments proximal or distal to the maximal lumen narrowing sites. The stenosis degree of BA was calculated by the ratio between the lumen diameter at the maximal lumen narrowing site and the reference luminal area (19, 20), which was classified as mild stenosis (< 50%), moderate stenosis (50–70%), and severe stenosis (70–99%). Plaque contrast enhancement was categorized based on black-blood MRI (grade 0, enhancement less than or equal to that of normal arterial walls seen elsewhere; grade I, enhancement greater than grade 0 but less than that of the pituitary infundibulum; and grade II, enhancement greater than or equal to that of the pituitary infundibulum) (21). IPH was defined as high signal intensity (150% intensity compared with the normal gray matter) on MP-RAGE images (20). The rupture of the fibrous cap was identified as the absence of the dark band between the lumen and the plaque core as well as the presence of a bright gray region adjacent to the lumen, corresponding to recent plaque hemorrhage or mural thrombus on 3D-MOTSA images (22). In our study, vulnerable plaque of the BA was defined as having one of the following characteristics (6): Severe stenosis, enhancement greater than or equal to grade I, IPH, or the rupture of the fibrous cap.

### Statistical Analysis

Continuous variables were expressed as means ± standard deviation (SD) or median (Q1–Q3). For the variables in both groups that exhibit a normal distribution, an independent *t*-test was used. For the variables that do not show a normal distribution, a non-parametric test was used. Categorical variables were expressed as count (percentage) and differences between groups were measured by the chi-square test.

Univariate analyses were performed to identify variables predictive of BA plaque and its numbers, as well as vulnerable plaque. Multivariable logistic regression models based on odds ratio (*OR*) with their associated 95% confidence interval (*CI*) were used to assess the relationship between hypertension and BA plaque, moderate or severe stenosis of BA plaque, and vulnerable plaque. A linear regression model was used to assess the relationship between hypertension and BA plaque numbers. The crude model was not adjusted for any confounder. The model I was only adjusted for age and sex. The model II was a confounder model. This confounder model screened covariates, such as age, sex, BMI, smoking and drinking status, T2DM, aspirin, statin, TC, TG, HDL, LDL, VLDL, Hcy, serum uric acid, and eGFR. We selected these confounders based on the fact that when they were included in this model, they changed the matched effect size by at least 10%. Table 1 shows the association of each confounder with the outcomes of interest. We considered the confounder model to be the main model.

**TABLE 1:** Univariate analyses for BA plaque and vulnerable plaque.

Covariate	Statistics	BA plaque		Number of BA plaque		Vulnerable plaque	
		*OR* (95%CI)	*P*-value	β (95%CI)	*P*-value	*OR* (95%CI)	*P*-value
Age	55.71 ± 12.58	1.06 (1.03, 1.09)	<0.001	0.03 (0.02, 0.04)	<0.001	1.03 (1.00, 1.06)	0.023
**Sex, n(%)**							
Male	133 (68.91%)	Ref		Ref		Ref	
Female	60 (31.09%)	0.76 (0.41, 1.42)	0.393	–0.18 (–0.57, 0.20)	0.349	0.61 (0.28, 1.33)	0.211
**Smoking status, n(%)**							
Never	125 (66.49%)	Ref		Ref		Ref	
Former smoker	10 (5.32%)	0.88 (0.24, 3.26)	0.844	0.02 (–0.80, 0.84)	0.962	1.71 (0.41, 7.10)	0.457
Current smoker	53 (28.19%)	1.09 (0.57, 2.08)	0.798	–0.13 (–0.53, 0.28)	0.549	1.05 (0.47, 2.32)	0.909
**Drinking status, n(%)**							
Never	147 (79.0%)	Ref		Ref		Ref	
Former drinker	10 (5.4%)	0.9 (0.2, 3.4)	0.893	–0.10 (–0.92, 0.71)	0.805	0.5 (0.1, 3.7)	0.460
Current drinker	29 (15.6%)	1.7 (0.8, 3.8)	0.201	0.37 (–0.14, 0.88)	0.154	1.8 (0.8, 4.4)	0.181
BMI (kg/m^2^)	25.77 ± 3.77	0.98 (0.90, 1.06)	0.618	–0.02 (–0.07, 0.03)	0.437	0.96 (0.86, 1.06)	0.403
**Hypertension classification, n (%)**							
No	66 (34.20%)	Ref		Ref		Ref	
Class I/II	47 (24.35%)	4.22 (1.85, 9.61)	<0.001	0.60 (0.15, 1.05)	0.010	3.82 (1.33, 10.98)	0.013
Class III	80 (41.45%)	5.29 (2.53, 11.08)	<0.001	0.81 (0.41, 1.20)	<0.001	4.29 (1.63, 11.26)	0.003
**T2DM, n(%)**							
No	136 (72.34%)	Ref		Ref		Ref	
Yes	52 (27.66%)	1.72 (0.90, 3.27)	0.099	0.30 (–0.10, 0.71)	0.141	1.35 (0.63, 2.87)	0.441
**Aspirin drug, n (%)**							
No	155 (81.58%)	Ref		Ref		Ref	
Yes	35 (18.42%)	0.96 (0.46, 2.01)	0.913	–0.06 (–0.52, 0.41)	0.807	1.14 (0.47, 2.74)	0.772
**Statins drugs, n (%)**							
No	157 (82.63%)	Ref		Ref		Ref	
Yes	33 (17.37%)	0.81 (0.38, 1.74)	0.585	–0.11 (–0.58, 0.37)	0.655	1.25 (0.52, 3.03)	0.622
TC (mmol/L)	4.07 ± 1.05	1.02 (0.77, 1.35)	0.891	0.07 (–0.10, 0.24)	0.426	0.84 (0.60, 1.19)	0.337
TG (mmol/L)	1.43 ± 0.93	0.84 (0.59, 1.18)	0.312	0.00 (–0.19, 0.20)	0.975	0.96 (0.65, 1.43)	0.852
HDL (mmol/L)	1.02 ± 0.24	1.14 (0.33, 3.87)	0.835	0.18 (–0.58, 0.94)	0.644	0.31 (0.06, 1.54)	0.151
LDL (mmol/L)	2.60 ± 0.80	0.98 (0.68, 1.42)	0.924	0.08 (–0.15, 0.30)	0.511	0.84 (0.53, 1.33)	0.456
VLDL (mmol/L)	0.46 ± 0.23	1.61 (0.45, 5.73)	0.459	0.35 (–0.44, 1.14)	0.382	0.78 (0.16, 3.78)	0.758
Hcy (μmol/L)	16.25 ± 11.23	0.99 (0.97, 1.02)	0.711	–0.01 (–0.02, 0.01)	0.411	0.97 (0.93, 1.02)	0.221
eGFR (ml/min)	94.62 ± 15.03	0.97 (0.95, 0.99)	0.004	–0.01 (–0.03, 0.00)	0.030	0.99 (0.97, 1.01)	0.297
SUA(mmol/L)	321.22 ± 100.84	1.00 (1.00, 1.00)	0.933	0.00 (0.00, 0.00)	0.942	1.00 (1.00, 1.01)	0.309

*Ref, reference; OR, odds ratio; β, effect size; CI, confidence interval; BA, basilar artery; BMI, body mass index; T2DM, type 2 diabetes mellitus; FBG, fasting blood glucose; TC, total cholesterol; TG, total triglyceride; HDL, high-density lipoprotein; LDL, low-density lipoprotein; VLDL, very low-density lipoprotein; Hcy, homocysteine; BUN, blood urea nitrogen; eGFR, estimated glomerular filtration rate; and SUA, serum uric acid.*

To ensure the robustness of data analysis, we also did the subgroup analyses that were performed using stratified multivariate regression and interaction analyses. Considering that there was a saturation effect of age on the prevalence of BA plaque or vulnerable plaque, the generalized additive model and smooth curve fitting (penalized spline method) were used to visually show the relationship between age and BA plaque and vulnerable plaque (Supplementary Figures 1, 2). If non-linearity was detected, we first use a recursive algorithm to calculate the inflection points and then construct a two-segment binary logistic regression model on both sides of the inflection points.

All statistical analyses were performed using the statistical package R (The R Foundation, version 3.4.3)^1^ and the Empower (R; X&Y Solutions, Inc., Boston, MA, United States).^2^ All values of *p* are two-tailed, and *p* < 0.05 was considered statistically significant.

## Results

### Clinical Characteristics of the Study Population

This study included 193 subjects (age: 55.71 ± 12.58 years, range 26–87 years; men, 68.91%), and the prevalence of hypertension was 65.80%. The clinical characteristics of participants are presented in Table 2 by the categories of hypertension classification. Compared with the normotensive group, patients with hypertension had a higher proportion of older adults, of aspirin and statins usage, and of BA plaque and vulnerable plaque, lower extents of eGFR, and more number of enhancements of BA plaques and serious plaque (all *p* < 0.05).

**TABLE 2:** Clinical characteristics of the participants grouped by the hypertension classification.

Variables	Hypertension classification			
	No (*n* = 66)	Class I/II (*n* = 47)	Class III (*n* = 80)	*P*-value
***General characteristics***				
Age (years)	50.40 ± 11.62	57.33 ± 12.28	58.98 ± 12.25	<0.001
Male, n (%)	49 (74.24%)	30 (63.83%)	54 (67.50%)	0.468
Smoking status, n (%)				0.491
Never	42 (64.62%)	33 (75.00%)	50 (63.29%)	
Former smoker	2 (3.08%)	2 (4.55%)	6 (7.59%)	
Current smoker	21 (32.31%)	9 (20.45%)	23 (29.11%)	
Drinking status, n (%)				0.556
Never	51 (80.95%)	37 (84.09%)	59 (74.68%)	
Former drinker	2 (3.17%)	3 (6.82%)	5 (6.33%)	
Current drinker	10 (15.87%)	4 (9.09%)	15 (18.99%)	
BMI (kg/m^2^)	25.51 ± 4.31	25.83 ± 3.27	25.94 ± 3.58	0.799
T2DM, n (%)	16 (24.24%)	12 (27.27%)	24 (30.77%)	0.682
Ischemic stroke or TIA, n (%)	21 (31.82%)	15 (34.09%)	32 (40.51%)	0.531
Impaired cognitive function, n (%)	1 (1.52%)	4 (9.09%)	5 (6.41%)	0.190
Aspirin, n (%)	6 (9.09%)	8 (18.18%)	21 (26.25%)	0.029
Statins, n (%)	6 (9.09%)	7 (15.91%)	20 (25.00%)	0.040
**Blood biochemical tests**				
FBG (mmol/L)	5.17 ± 1.28	5.28 ± 1.48	5.77 ± 2.21	0.107
TC (mmol/L)	4.00 ± 1.01	3.95 ± 1.06	4.21 ± 1.07	0.343
TG (mmol/L)	1.27 (0.90–1.82)	1.07 (0.87–1.48)	1.20 (0.86–1.74)	0.965
HDL (mmol/L)	0.98 ± 0.24	0.99 ± 0.21	1.05 ± 0.24	0.169
LDL (mmol/L)	2.56 ± 0.76	2.49 ± 0.87	2.69 ± 0.78	0.372
VLDL (mmol/L)	0.46 (0.33–0.55)	0.40 (0.27–0.59)	0.42 (0.32–0.59)	0.984
Hcy (μmol/L)	12.80 (10.70–16.00)	13.40 (10.90–18.65)	12.90 (10.45–17.75)	0.266
BUN (mmol/L)	4.74 ± 1.34	4.76 ± 1.38	5.29 ± 2.41	0.152
Serum creatinine (mmol/L)	70.14 ± 10.96	71.74 ± 14.30	78.12 ± 32.75	0.102
eGFR (ml/min)	101.39 ± 11.11	94.60 ± 13.44	89.08 ± 16.47	<0.001
Serum uric acid (mmol/L)	310.08 ± 94.31	317.13 ± 104.76	332.66 ± 103.84	0.397
CK-MB (μg/L)	16.09 ± 5.46	15.59 ± 3.64	15.63 ± 3.81	0.786
***Plaque characteristics***				
BA plaque, n (%)	14 (21.21%)	25 (53.19%)	47 (58.75%)	<0.001
Number of BA plaque, n (%)				0.003
Zero	52 (78.79%)	22 (46.81%)	33 (41.25%)	
One	9 (13.64%)	14 (29.79%)	24 (30.00%)	
Two	2 (3.03%)	6 (12.77%)	10 (12.50%)	
Three	0 (0.00%)	0 (0.00%)	1 (1.25%)	
Four	3 (4.55%)	5 (10.64%)	12 (15.00%)	
Vulnerable plaque, n (%)	6 (9.09%)	13 (27.66%)	24 (30.00%)	0.006
Stenosis of BA plaque, n (%)				<0.001
No	52 (78.79%)	26 (55.32%)	33 (41.25%)	
Mild	10 (15.15%)	17 (36.17%)	32 (40.00%)	
Moderate	3 (4.55%)	1 (2.13%)	6 (7.50%)	
Severe	1 (1.52%)	3 (6.38%)	9 (11.25%)	
Enhancing degree of BA plaque, n (%)				0.010
No	64 (96.97%)	35 (74.47%)	69 (86.25%)	
Mild	1 (1.52%)	10 (21.28%)	8 (10.00%)	
Severe	1 (1.52%)	2 (4.26%)	3 (3.75%)	
Intraplaque hemorrhage of BA plaque, n (%)	3 (4.55%)	1 (2.13%)	10 (12.50%)	0.054
Lipid fiber cap rupture of BA plaque, n (%)	1 (1.52%)	1 (2.13%)	4 (5.00%)	0.437

*BMI, body mass index; T2DM, type 2 diabetes mellitus; TIA, transient ischemic attacks; FBG, fasting blood glucose; TC, total cholesterol; TG, total triglyceride; HDL, high-density lipoprotein; LDL, low-density lipoprotein; VLDL, very low-density lipoprotein; Hcy, homocysteine; BUN, blood urea nitrogen; eGFR, estimated glomerular filtration rate; CK-MB, creatine kinase-MB; and BA, basilar artery.*

### Univariate Analyses for Basilar Artery Plaque and Vulnerable Plaque

Univariate analyses found that older adults, patients with hypertension, and those with lower eGFR increased the risk for BA plaque and its numbers, while older adults and patients with hypertension were associated with the risk of vulnerable plaque (Table 1).

### Relationship Between Hypertension and Basilar Artery Plaque and Vulnerable Plaque

Multivariable analyses indicated that patients with hypertension had an increased risk for BA plaque and its numbers (adjusted-*OR*: 4.32, 95% *CI* 1.89–9.88, *p* < 0.001; adjusted-β: 0.55, 95% *CI* 0.14–0.96, *p* = 0.009, respectively) and had a higher proportion of moderate or severe stenosis of BA plaque and vulnerable plaque (adjusted-*OR*: 3.08, 95% *CI* 0.77–12.32, *p* = 0.111; adjusted-*OR*: 4.52, 95% *CI* 1.50–13.64, *p* = 0.007, respectively; Table 3) compared with the normotensive group. When the normotensive group was used as reference, individuals with tertiary hypertension had an increased risk for BA plaque and its numbers (adjusted-*OR*: 4.83, 95% *CI* 1.96–11.88, *p* < 0.001; adjusted-β: 0.63, 95% *CI* 0.18–1.08, *p* = 0.007, respectively) and had a higher proportion of moderate or severe stenosis of BA plaque and vulnerable plaque (adjusted-*OR*: 5.88, 95% *CI* 1.31–26.46, *p* = 0.021; adjusted-*OR*: 5.44, 95% *CI* 1.67–17.74, *p* = 0.005, respectively).

**TABLE 3:** Relationship between hypertension and BA plaque and vulnerable plaque in different models.

Variable	Event, n (%)	Crude model		Model I		Model II	
BA plaque		*OR* (95%CI)	*P*-value	*OR* (95%CI)	*P*-value	*OR* (95%CI)	*P*-value
**Hypertension**							
No	14 (16.28%)	Ref		Ref		Ref	
Yes	72 (83.72%)	4.86 (2.45, 9.66)	<0.001	3.71 (1.79, 7.68)	<0.001	4.32 (1.89, 9.88)	<0.001
**Hypertension classification**							
No	14 (16.28%)	Ref		Ref		Ref	
Class I/II	25 (29.07%)	4.22 (1.85, 9.61)	<0.001	3.49 (1.46, 8.35)	0.005	3.71 (1.42, 9.69)	0.007
Class III	47 (54.65%)	5.29 (2.53, 11.08)	<0.001	3.85 (1.76, 8.40)	<0.001	4.83 (1.96, 11.88)	<0.001
**Number of BA plaque**		**β (95%CI)**	***P*-value**	**β (95%CI)**	***P*-value**	**β (95%CI)**	***P*-value**
**Hypertension**							
No	–	Ref		Ref		Ref	
Yes	–	0.73 (0.37, 1.09)	<0.001	0.55 (0.17, 0.92)	0.005	0.55 (0.14, 0.96)	0.009
**Hypertension classification**							
No	–	Ref		Ref		Ref	
Class I/II	–	0.60 (0.15, 1.05)	0.010	0.46 (–0.01, 0.92)	0.054	0.44 (–0.05, 0.92)	0.078
Class III	–	0.81 (0.41, 1.20)	<0.001	0.60 (0.19, 1.01)	0.005	0.63 (0.18, 1.08)	0.007
**Moderate-severe stenosis of BA plaque**		***OR* (95%CI)**	***P*-value**	***OR* (95%CI)**	***P*-value**	***OR* (95%CI)**	***P*-value**
**Hypertension**							
No	4 (17.39%)	Ref		Ref		Ref	
Yes	19 (82.61%)	2.73 (0.89, 8.38)	0.080	2.00 (0.62, 6.44)	0.247	3.08 (0.77, 12.32)	0.111
**Hypertension classification**							
No	4 (17.39%)	Ref		Ref		Ref	
Class I/II	4 (17.39%)	1.44 (0.34, 6.08)	0.618	1.10 (0.25, 4.83)	0.896	0.84 (0.12, 5.87)	0.863
Class III	15 (65.22%)	3.58 (1.13, 11.37)	0.031	2.60 (0.78, 8.69)	0.122	5.88 (1.31, 26.46)	0.021
**Vulnerable plaque**		***OR* (95%CI)**	***P*-value**	***OR* (95%CI)**	***P*-value**	***OR* (95%CI)**	***P*-value**
**Hypertension**							
No	6 (13.95%)	Ref		Ref		Ref	
Yes	37 (86.05%)	4.11 (1.63, 10.34)	0.003	3.55 (1.36, 9.24)	0.009	4.52 (1.50, 13.64)	0.007
**Hypertension classification**							
No	6 (13.95%)	Ref		Ref		Ref	
Class I/II	13 (30.23%)	3.82 (1.33, 10.98)	0.013	3.46 (1.17, 10.27)	0.025	3.53 (1.02, 12.27)	0.047
Class III	24 (55.81%)	4.29 (1.63, 11.26)	0.003	3.60 (1.32, 9.82)	0.012	5.44 (1.67, 17.74)	0.005

*BA, basilar artery; Ref, reference; OR, odds ratio; β, effect size; and CI, confidence interval.*

*Model I adjusted for age and sex.*

*Model II adjusted for age, sex, smoking. and drinking status, T2DM, LDL, and eGFR.*

### Subgroup Analyses Between Hypertension and Basilar Artery Plaque and Vulnerable Plaque

To explore whether this positive association between hypertension and BA plaque was still stable in different subgroups, we conducted stratified and interaction analyses. Subgroup analyses showed that older adults, men, patients with T2DM, and those with hyper-homocysteinemia (HHcy was defined as Hcy ≥ 15 μmol/L) increased the *OR*s of hypertension on the risk for BA plaque (Table 4) and vulnerable plaque (Table 5). In terms of sex differences, we found that male participants were more likely to smoke and drink and had lower extents of HDL and higher levels of Hcy, serum uric acid, and creatinine compared with female subjects (all *p* < 0.05, Supplementary Table 1). Furthermore, there were no significant interactions between hypertension and BA plaque as well as vulnerable plaque in any of the subgroups, such as age (< 40, 40–49, 50–59, and ≥ 60 years old), sex (male vs. female), T2DM (no vs. yes), HHcy (no vs. yes) (all *p* for interactions > 0.05).

**TABLE 4:** Odds ratios of hypertension on BA plaque in the prespecified and exploratory subgroups.

Characteristic	No. of participants	Event, n (%)	*OR* (95%CI)	*P*-value	*P*-value for interaction
Age (years)					0.897
<40	24	2 (2.33%)	^§^		
40–49	36	12 (13.95%)	2.10 (0.14, 31.91)	0.593	
50–59	50	23 (26.74%)	8.05 (1.04, 62.41)	0.046	
≥60	79	49 (56.98%)	4.44 (0.82, 23.94)	0.083	
Sex					0.664
Male	133	62 (72.09%)	4.82 (1.59, 14.58)	0.005	
Female	60	24 (27.91%)	2.14 (0.23, 19.58)	0.501	
T2DM					0.456
No	136	55 (66.27%	2.89 (0.94, 8.89)	0.065	
Yes	52	28 (33.73%)	6.19 (0.85, 45.28)	0.073	
Hcy (μmol/L)					0.429
<15	112	48 (64.00%)	2.63 (0.90, 7.72)	0.078	
≥15	59	27 (36.00%)	6.29 (0.88, 44.89)	0.067	

*BA, basilar artery; OR, odds ratio; CI, confidence interval; T2DM, type 2 diabetes mellitus; and Hcy, homocysteine.*

*Each stratification adjusted for age, sex, T2DM, LDL, eGFR, BMI, Hcy, aspirin, and statins usage except for the subgroup variable.*

*^§^The model failed because of the small sample size.*

**TABLE 5:** Odds ratios of hypertension on BA vulnerable plaque in the prespecified and exploratory subgroups.

Characteristics	No. of participants	Event, n (%)	*OR* (95%CI)	*P*-value	*P*-value for interaction
Age (years)					0.140
<40	24	2 (4.65%)	^§^	0.999	
40–49	36	3 (6.98%)	1.70 (0.12, 23.73)	0.695	
50–59	50	13 (30.23%)	12.65 (1.23, 129.76)	0.033	
≥ 60	79	25 (58.14%)	3.30 (0.71, 15.30)	0.128	
Sex					0.602
Male	133	33 (76.74%)	6.90 (1.86, 25.59)	0.004	
Female	60	10 (23.26%)	3.15 (0.24, 41.34)	0.382	
T2DM					0.714
No	136	27 (67.50%)	3.47 (0.80, 15.02)	0.096	
Yes	52	13 (32.50%)	5.30 (0.53, 53.27)	0.157	
Hcy (μmol/L)					0.690
<15	112	23 (63.89%)	3.42 (0.88, 13.36)	0.076	
≥15	59	13 (36.11%)	6.24 (0.43, 90.98)	0.181	

*BA, basilar artery; OR, odds ratio; CI, confidence interval; T2DM, type 2 diabetes mellitus; and Hcy, homocysteine.*

*Each stratification adjusted for age, sex, BMI, T2DM, Hcy, and eGFR except for the subgroup variable.*

*^§^The model failed because of the small sample size.*

### Saturation Effect Analysis of Age on the Prevalence of Basilar Artery Plaque or Vulnerable Plaque

Stratified analyses found that compared with the age group of 50–59 years old, subjects with age older than 60 years old had a reduced effect size of hypertension on the risk for BA vulnerable plaque (adjusted-*OR*: 8.05, 95% *CI* 1.04–62.41, *p* = 0.046 vs. adjusted-*OR*: 4.44, 95% *CI* 0.82–23.94, *p* = 0.083; Table 4) and vulnerable plaque (adjusted-*OR*: 12.65, 95% *CI* 1.23–129.76, *p* = 0.033 vs. adjusted-*OR*: 3.30, 95% *CI* 0.71–15.30, *p* = 0.128; Table 5). The clinical characteristics of participants grouped by age are presented in Supplementary Table 2.

Therefore, we used the generalized additive model and penalized spline method to assess whether there was a non-linear relationship between age and the prevalence of BA plaque (Supplementary Figure 1) or vulnerable plaque (Supplementary Figure 2). In the adjusted smoothing curve, the relationship between age and the prevalence of BA plaque and vulnerable plaque was not linear. With the increase of age, the prevalence of BA plaque and vulnerable plaque increased first and then leveled off. Visual inspection shows that the inflection point is approximately 60 years old. We further fitted the association between age and the prevalence of BA plaque and vulnerable plaque using the two-piecewise binary logistic regression model. The inflection points of age were 61 (Supplementary Table 3) and 60 (Supplementary Table 4) years old. The effect size [*OR* (95% *CI*)] of age on the prevalence of BA plaque was 1.10 (1.05, 1.16) on the left side and 0.99 (0.92, 1.06) on the right of the inflection point, meanwhile the effect size [*OR* (95% *CI*)] of age on the prevalence of vulnerable plaque was 1.09 (1.02, 1.15) on the left side and 0.97 (0.90, 1.04) on the right of the inflection point. These results suggested that there was a saturation effect of age on the prevalence of BA plaque and vulnerable plaque.

## Discussion

Digital subtraction angiography is the most accurate and reliable diagnostic method currently, but it is an invasive procedure and cannot reflect the pathological characteristics of the intracranial artery wall. In recent years, HR-MRI imaging has been developed rapidly and has become a useful auxiliary tool of imaging technology. HR-MRI has an outstanding effect in differentiating atherosclerotic plaque, vasculitis, reversible cerebral vasoconstriction syndrome, and arterial dissection (3–8). Compared with other traditional lumen imaging techniques, HR-MRI has been found to be more accurate in determining the extent of atherosclerotic lesions (23, 24). Klein et al. (25) studied 41 patients with pontine infarction and found that HR-MRI was superior to TOF MRA in detecting basilar atherosclerotic lesions. For example, 77% of patients with paramedian wave infarction had evidence of BA plaque on HR-MRI, while 65% of these patients had normal TOF MRA. In ischemic stroke patients with symptomatic BA stenosis, HR-MRI was used to identify IPH or dissection (26). HR-MRI results enabled underlying symptomatic branch atheromatous disease to be detected in patients with lacunar infarction using normal angiography (27). Since basilar stenosis may be underestimated by MRA, HR-MRI may provide additional information for predicting progressive motor deficits and evaluating BA stenosis in patients with acute unilateral pontine infarction (28).

This study found that hypertension was the independent risk factor for BA plaque and vulnerable plaque evaluated by HR-MRI in the Chinese Han population. HR-MRI is an effective tool for identifying stroke etiology in patients with non-stenotic intracranial arteries. Detection of the vessel wall of arteries, such as the middle cerebral artery (MCA) and BA, might improve the ability to identify advanced but unrecognized intracranial atherosclerotic lesions (6). In 15 patients with an acute lacunar infarct but normal angiography findings without apparent cause of stroke, HR-MRI demonstrated enhancing atherosclerotic plaque in the MCA or BA in 9 (60%) patients (27). Similar studies have shown HR-MRI evidence of atheromatous plaque in the MCA of 52 or 46.9% of patients with MCA territory lacunar infarcts or single lenticulostriate infarction and in the BA of 42% of patients with pontine infarcts but normal MRA findings (25, 29, 30). Patients with HR-MRI identified plaque presented larger infarction lesions and more proximal lesions than patients without plaque (25).

The etiology of stenosis or occlusion was unclear until the development of HR-MRI. With HR-MRI, stroke etiology is better understood, and factors affecting each etiology can be identified. In Chinese patients with ischemic stroke, hypertension is a risk factor for intracranial artery stenosis and posterior circulation artery stenosis (31). Patients with BA plaque with IPH were older and had a higher prevalence of hypertension and hyperlipidemia than the other patients (26). Metabolic syndrome (MetS) and diabetes mellitus along with hypertension are associated with more extensive ICAS than MetS and hypertension or MetS alone (32). Lifestyle changes and aggressive management of atherosclerotic risk factors, such as hypertension, are vital for preventing the occurrence of stroke.

There were some limitations in our study. A relatively small sample size would affect the generalization ability of the model. Second, our research lacked the dynamic and longitudinal HR-MRI evaluation.

In conclusion, we found that hypertension was the independent risk factor of BA plaque and vulnerable plaque evaluated by HR-MRI in the Chinese Han population.

## Data Availability Statement

The original contributions presented in the study are included in the article/Supplementary Material, further inquiries can be directed to the corresponding author/s.

## Ethics Statement

The studies involving human participants were reviewed and approved by the Institutional Ethics Committees of the Second Affiliated Hospital of Nanchang University. The patients/participants provided their written informed consent to participate in this study.

## Author Contributions

FH: conceptualization, methodology, research investigation, and writing–original draft preparation. FL: data curation. HX: software. MD: visualization. YX: supervision, writing–reviewing and editing. All authors read and approved the final version of the manuscript.

## Conflict of Interest

The authors declare that the research was conducted in the absence of any commercial or financial relationships that could be construed as a potential conflict of interest.

## Publisher’s Note

All claims expressed in this article are solely those of the authors and do not necessarily represent those of their affiliated organizations, or those of the publisher, the editors and the reviewers. Any product that may be evaluated in this article, or claim that may be made by its manufacturer, is not guaranteed or endorsed by the publisher.

## Footnotes

1
http://www.R-project.org

2
www.empowerstats.com

## Supplementary Material

Supplementary Figure 1 The smooth curve of correlation between age and BA plaque. Adjust for drinking status, hypertension, and type 2 diabetes mellitus (T2DM). BA, basilar artery.

Supplementary Figure 2 The smooth curve of correlation between age and BA vulnerable plaque. Adjust for body mass index (BMI), drinking status, hypertension, T2DM, and serum uric acid. BA, basilar artery.
