# Supplementary material for: Relationship Between Hypertension and Basilar Atherosclerosis in Chinese Han Population: A High-Resolution Magnetic Resonance Imaging Study
Source: Front Cardiovasc Med. 2022 Apr 27;9:830664. doi: 10.3389/fcvm.2022.830664 (PMC9094699; doi:10.3389/fcvm.2022.830664)
Supplement: Supplementary file 1 [file Data_Sheet_1.doc]

**Supplementary Tables**

| **Supplementary Table 1. Clinical characteristics of participants grouping by sex.** | | | |
| --- | --- | --- | --- |
| **Variables** | **Male（n=133）** | **Female（n=60）** | ***P*-value** |
| *General Characteristics* |  |  |  |
| Age (years) | 55.12 ± 12.66 | 57.09 ± 12.42 | 0.325 |
| Smoking status, n(%) |  |  | <0.001 |
| Never | 67 (51.54%) | 58 (100.00%) |  |
| Former smoker | 10 (7.69%) | 0 (0.00%) |  |
| Current smoker | 53 (40.77%) | 0 (0.00%) |  |
| Drinking status, n(%) |  |  | <0.001 |
| Never | 89 (69.53%) | 58 (100.00%) |  |
| Former drinker | 10 (7.81%) | 0 (0.00%) |  |
| Current drinker | 29 (22.66%) | 0 (0.00%) |  |
| BMI (kg/m^2) | 25.96 ± 3.84 | 25.35 ± 3.60 | 0.321 |
| Hypertension, n(%) | 84 (63.16%) | 43 (71.67%) | 0.249 |
| T2DM, n(%) | 37 (28.46%) | 15 (25.86%) | 0.713 |
| Ischemic stroke or TIA, n(%) | 49 (37.40%) | 19 (32.76%) | 0.539 |
| Impaired cognitive function, n(%) | 5 (3.85%) | 5 (8.62%) | 0.178 |
| Aspirin, n(%) | 23 (17.56%) | 12 (20.34%) | 0.647 |
| Statins, n(%) | 23 (17.56%) | 10 (16.95%) | 0.918 |
| *Blood Biochemicaltests* |  |  |  |
| FBG (mmol/L) | 5.40 ± 1.86 | 5.55 ± 1.61 | 0.594 |
| TC (mmol/L) | 4.06 ± 1.04 | 4.11 ± 1.08 | 0.729 |
| TG (mmol/L) | 1.45 ± 1.01 | 1.40 ± 0.73 | 0.771 |
| HDL (mmol/L) | 0.98 ± 0.22 | 1.09 ± 0.25 | 0.002 |
| LDL (mmol/L) | 2.60 ± 0.79 | 2.59 ± 0.81 | 0.914 |
| VLDL (mmol/L) | 0.48 ± 0.24 | 0.43 ± 0.21 | 0.210 |
| Hcy (μmol/L) | 13.50 (11.28-19.35) | 11.20 (9.15-13.80) | <0.001 |
| BUN (mmol/L) | 5.21 ± 2.09 | 4.48 ± 1.19 | 0.013 |
| Serum creatinine (mmol/L) | 78.78 ± 25.68 | 63.27 ± 12.25 | <0.001 |
| eGFR (ml/min) | 95.01 ± 15.36 | 93.77 ± 14.36 | 0.601 |
| Serum uric acid (mmol/L) | 341.23 ± 99.44 | 278.15 ± 90.51 | <0.001 |
| CK-MB (ug/L) | 16.07 ± 4.84 | 15.15 ± 3.19 | 0.182 |
| *Plaque characteristics* |  |  |  |
| BA plaque, n(%) | 62 (46.62%) | 24 (40.00%) | 0.392 |
| Number of BA plaque, n(%) |  |  | 0.869 |
| Zero | 71 (53.38%) | 36 (60.00%) |  |
| One | 33 (24.81%) | 14 (23.33%) |  |
| Two | 13 (9.77%) | 5 (8.33%) |  |
| Three | 1 (0.75%) | 0 (0.00%) |  |
| Four | 15 (11.28%) | 5 (8.33%) |  |
| Vulnerable plaque, n(%) | 33 (24.81%) | 10 (16.67%) | 0.208 |
| Stenosis of BA plaque, n(%) |  |  | 0.449 |
| No | 75 (56.39%) | 36 (60.00%) |  |
| Mild | 44 (33.08%) | 15 (25.00%) |  |
| Moderate | 5 (3.76%) | 5 (8.33%) |  |
| Severe | 9 (6.77%) | 4 (6.67%) |  |
| Enhancing degree of BA plaque, n(%) |  |  | 0.217 |
| No | 112 (84.21%) | 56 (93.33%) |  |
| Mild | 16 (12.03%) | 3 (5.00%) |  |
| Severe | 5 (3.76%) | 1 (1.67%) |  |
| Intraplaque hemorrhage of BA plaque, n(%) | 10 (7.52%) | 4 (6.67%) | 0.833 |
| Iipid fiber cap rupture of BA plaque, n(%) | 4 (3.01%) | 2 (3.33%) | 0.904 |
| Abbreviations: BMI, body mass index; T2DM, type 2 diabetes mellitus; TIA, transient ischemic attacks; FBG, fasting blood glucose; TC, total cholesterol; TG, total triglyceride; HDL, high-density lipoprotein; LDL, low-density lipoprotein; VLDL, very low-density lipoprotein; Hcy, homocysteine; BUN, blood urea nitrogen; eGFR, estimated glomerular filtration rate; CK-MB, creatine kinase-MB; BA, basilar artery. | | | |

| **Supplementary Table 2. Clinical characteristics of participants grouping by age.** | | | | | |
| --- | --- | --- | --- | --- | --- |
| **Variables** | **Age classification** | | | | |
|  | **<40ys (n=24)** | **40-49ys (n=36)** | **50-59ys (n=50)** | **≥60ys (n=79)** | ***P*-value** |
| *General Characteristics* |  |  |  |  |  |
| Age (years) | 34.12 ± 3.60 | 45.92 ± 2.42 | 54.44 ± 2.62 | 67.54 ± 6.36 | <0.001 |
| Male, n(%) | 16 (66.67%) | 30 (83.33%) | 35 (70.00%) | 51 (64.56%) | 0.233 |
| Smoking status, n(%) |  |  |  |  | 0.122 |
| Never | 17 (70.83%) | 19 (52.78%) | 34 (70.83%) | 52 (67.53%) |  |
| Former smoker | 0 (0.00%) | 1 (2.78%) | 5 (10.42%) | 4 (5.19%) |  |
| Current smoker | 7 (29.17%) | 16 (44.44%) | 9 (18.75%) | 21 (27.27%) |  |
| Drinking status, n(%) |  |  |  |  | 0.136 |
| Never | 18 (75.00%) | 23 (65.71%) | 40 (83.33%) | 63 (82.89%) |  |
| Former drinker | 0 (0.00%) | 2 (5.71%) | 3 (6.25%) | 5 (6.58%) |  |
| Current drinker | 6 (25.00%) | 10 (28.57%) | 5 (10.42%) | 8 (10.53%) |  |
| BMI (kg/m^2) | 27.62 ± 6.12 | 26.16 ± 3.97 | 25.65 ± 3.04 | 25.12 ± 3.09 | 0.054 |
| Hypertension, n(%) | 8 (33.33%) | 24 (66.67%) | 32 (64.00%) | 62 (78.48%) | <0.001 |
| T2DM, n(%) | 2 (8.33%) | 8 (22.86%) | 13 (27.08%) | 28 (36.36%) | 0.050 |
| Ischemic stroke or TIA, n(%) | 5 (20.83%) | 14 (38.89%) | 20 (41.67%) | 29 (37.66%) | 0.360 |
| Impaired cognitive function, n(%) | 0 (0.00%) | 1 (2.86%) | 4 (8.33%) | 5 (6.49%) | 0.426 |
| Aspirin, n(%) | 2 (8.33%) | 12 (33.33%) | 12 (25.00%) | 9 (11.54%) | 0.014 |
| Statins, n(%) | 3 (12.50%) | 11 (30.56%) | 8 (16.67%) | 11 (14.10%) | 0.155 |
| *Blood Biochemicaltests* |  |  |  |  |  |
| FBG (mmol/L) | 4.45 ± 0.64 | 5.73 ± 2.57 | 5.50 ± 1.59 | 5.60 ± 1.64 | 0.030 |
| TC (mmol/L) | 4.19 ± 1.05 | 4.27 ± 1.23 | 3.93 ± 0.91 | 4.02 ± 1.06 | 0.486 |
| TG (mmol/L) | 1.55 ± 0.76 | 1.76 ± 1.54 | 1.38 ± 0.65 | 1.27 ± 0.70 | 0.068 |
| HDL (mmol/L) | 0.94 ± 0.20 | 1.03 ± 0.20 | 1.00 ± 0.23 | 1.03 ± 0.26 | 0.397 |
| LDL (mmol/L) | 2.74 ± 0.79 | 2.70 ± 0.98 | 2.51 ± 0.70 | 2.55 ± 0.78 | 0.553 |
| VLDL (mmol/L) | 0.51 ± 0.24 | 0.54 ± 0.30 | 0.43 ± 0.21 | 0.44 ± 0.20 | 0.105 |
| Hcy (μmol/L) | 19.61 ± 13.28 | 16.83 ± 11.22 | 17.54 ± 14.72 | 14.31 ± 7.38 | 0.204 |
| BUN (mmol/L) | 4.24 ± 1.24 | 5.51 ± 2.16 | 5.19 ± 2.37 | 4.85 ± 1.52 | 0.057 |
| Serum creatinine (mmol/L) | 69.10 ± 11.51 | 84.51 ± 45.24 | 71.17 ± 11.63 | 72.49 ± 14.11 | 0.091 |
| eGFR (ml/min) | 112.97 ± 8.66 | 96.86 ± 19.10 | 96.25 ± 8.33 | 86.42 ± 11.63 | <0.001 |
| Serum uric acid (mmol/L) | 360.49 ± 101.90 | 358.01 ± 113.14 | 325.71 ± 105.95 | 289.17 ± 82.46 | <0.001 |
| CK-MB (ug/L) | 15.28 ± 3.02 | 17.14 ± 7.33 | 15.72 ± 3.57 | 15.50 ± 3.23 | 0.265 |
| *Plaque characteristics* |  |  |  |  |  |
| BA plaque, n(%) | 2 (8.33%) | 12 (33.33%) | 23 (46.00%) | 49 (62.03%) | <0.001 |
| Number of BA plaque, n(%) |  |  |  |  | <0.001 |
| Zero | 22 (91.67%) | 24 (66.67%) | 27 (54.00%) | 30 (37.97%) |  |
| One | 0 (0.00%) | 10 (27.78%) | 14 (28.00%) | 23 (29.11%) |  |
| Two | 2 (8.33%) | 1 (2.78%) | 4 (8.00%) | 11 (13.92%) |  |
| Three | 0 (0.00%) | 0 (0.00%) | 1 (2.00%) | 0 (0.00%) |  |
| Four | 0 (0.00%) | 1 (2.78%) | 4 (8.00%) | 15 (18.99%) |  |
| Vulnerable plaque, n(%) | 2 (8.33%) | 3 (8.33%) | 13 (26.00%) | 25 (31.65%) | 0.012 |
| Stenosis of BA plaque, n(%) |  |  |  |  | <0.001 |
| No | 22 (91.67%) | 25 (69.44%) | 28 (56.00%) | 32 (40.51%) |  |
| Mild | 1 (4.17%) | 11 (30.56%) | 16 (32.00%) | 31 (39.24%) |  |
| Moderate | 0 (0.00%) | 0 (0.00%) | 4 (8.00%) | 6 (7.59%) |  |
| Severe | 1 (4.17%) | 0 (0.00%) | 2 (4.00%) | 10 (12.66%) |  |
| Enhancing degree of BA plaque, n(%) |  |  |  |  | 0.226 |
| No | 24 (100.00%) | 33 (91.67%) | 42 (84.00%) | 65 (82.28%) |  |
| Mild | 0 (0.00%) | 2 (5.56%) | 5 (10.00%) | 12 (15.19%) |  |
| Severe | 0 (0.00%) | 1 (2.78%) | 3 (6.00%) | 2 (2.53%) |  |
| Intraplaque hemorrhage of BA plaque, n(%) | 1 (4.17%) | 0 (0.00%) | 4 (8.00%) | 9 (11.39%) | 0.164 |
| Iipid fiber cap rupture of BA plaque, n(%) | 0 (0.00%) | 0 (0.00%) | 1 (2.00%) | 5 (6.33%) | 0.191 |
| Abbreviations: BMI, body mass index; T2DM, type 2 diabetes mellitus; TIA, transient ischemic attacks; FBG, fasting blood glucose; TC, total cholesterol; TG, total triglyceride; HDL, high-density lipoprotein; LDL, low-density lipoprotein; VLDL, very low-density lipoprotein; Hcy, homocysteine; BUN, blood urea nitrogen; eGFR, estimated glomerular filtration rate; CK-MB, creatine kinase-MB; BA, basilar artery. | | | | | |

| **Supplementary Table 3. Saturation effect analysis of age and BA plaque using Piece-wise logistic regression.** | | | | | | |
| --- | --- | --- | --- | --- | --- | --- |
| Inflection point of age | Number of participants | Event, n(%) | Effect size (*OR*) | *95%CI* | *P*-value | LLR test |
| <61years | 107 | 28 (37.84%) | 1.10 | (1.05, 1.16) | <0.001 | 0.030 |
| ≥61years | 86 | 46 (62.16%) | 0.99 | (0.92, 1.06) | 0.693 |
| Effect：BA plaque; Cause: age.  Adjusted for drinking status, hypertension and T2DM. Abbreviations: BA, basilar artery; *OR*, odds ratio; *CI*, confidence interval; LLR, log-likelihood ratio. | | | | | | |
|

| **Supplementary Table 4. Saturation effect analysis of age and BA vulnerable plaque using Piece-wise logistic regression.** | | | | | | |
| --- | --- | --- | --- | --- | --- | --- |
| Inflection point of age | Number of participants | Event, n(%) | Effect size (*OR*) | *95%CI* | P-value | LLR test |
| <60years | 150 | 54 (68.35%) | 1.09 | (1.02, 1.15) | 0.006 | 0.035 |
| ≥60years | 43 | 25 (31.65%) | 0.97 | (0.90, 1.04) | 0.386 |
| Effect: BA vulnerable plaque; Cause: age.  Adjusted for BMI, drinking status, hypertension, T2DM and serum uric acid. Abbreviations: BA, basilar artery; *OR*, odds ratio; *CI*, confidence interval; LLR, log-likelihood ratio. | | | | | | |
|
