# Supplementary figures and images for: Relationship Between Hypertension and Basilar Atherosclerosis in Chinese Han Population: A High-Resolution Magnetic Resonance Imaging Study
Source: Front Cardiovasc Med. 2022 Apr 27;9:830664. doi: 10.3389/fcvm.2022.830664 (PMC9094699; doi:10.3389/fcvm.2022.830664)

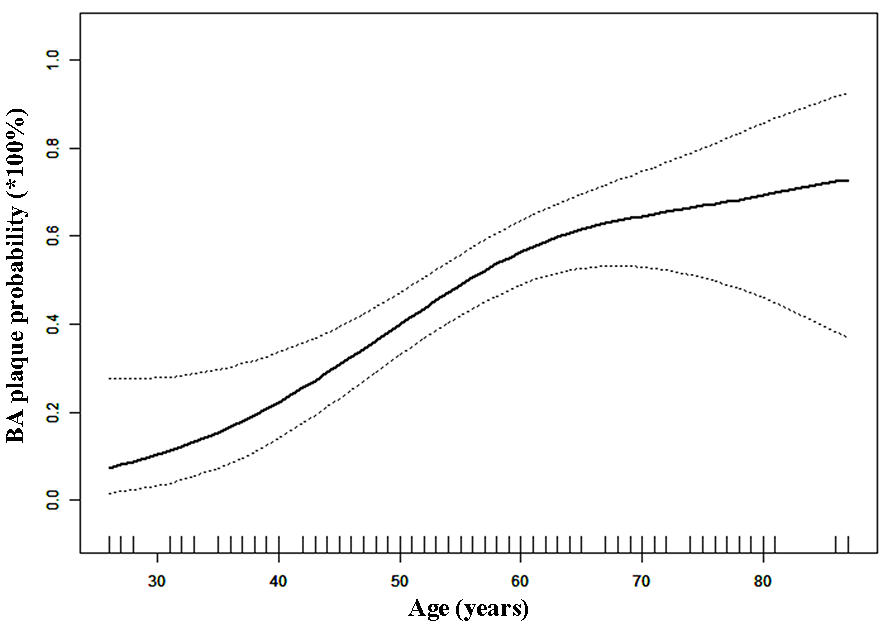

Supplement: Supplementary Figure 1 — The smooth curve of correlation between age and BA plaque. Adjust for drinking status, hypertension, and type 2 diabetes mellitus (T2DM). BA, basilar artery. [file Image_1.TIF]

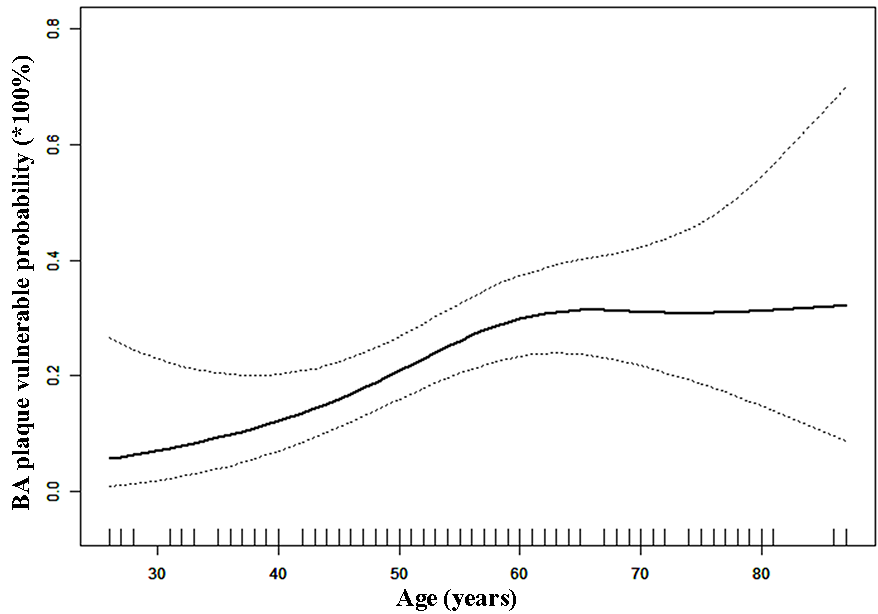

Supplement: Supplementary Figure 2 — The smooth curve of correlation between age and BA vulnerable plaque. Adjust for body mass index (BMI), drinking status, hypertension, T2DM, and serum uric acid. BA, basilar artery. [file Image_2.TIF]
